# Subject-independent emotion recognition based on physiological signals: a three-stage decision method

**DOI:** 10.1186/s12911-017-0562-x

**Published:** 2017-12-20

**Authors:** Jing Chen, Bin Hu, Yue Wang, Philip Moore, Yongqiang Dai, Lei Feng, Zhijie Ding

**Affiliations:** 1 F. Joseph Halcomb III, M.D. Department of Biomedical Engineering, University of Kentucky, Lexington, 40506 USA; 2 School of Information Science and Engineering, Lanzhou University, Lanzhou, 730000 China; 3 Beijing Anding Hospital of Capital Medical University, Beijing, 100088 China; 4 The Third People’s Hospital of Tianshui, Tianshui, 741020 China

**Keywords:** Emotion recognition, Multimodal physiological signals, Subject-independent, Stage-divided

## Abstract

**Background:**

Collaboration between humans and computers has become pervasive and ubiquitous, however current computer systems are limited in that they fail to address the emotional component. An accurate understanding of human emotions is necessary for these computers to trigger proper feedback. Among multiple emotional channels, physiological signals are synchronous with emotional responses; therefore, analyzing physiological changes is a recognized way to estimate human emotions. In this paper, a three-stage decision method is proposed to recognize four emotions based on physiological signals in the multi-subject context. Emotion detection is achieved by using a stage-divided strategy in which each stage deals with a fine-grained goal.

**Methods:**

The decision method consists of three stages. During the training process, the initial stage transforms mixed training subjects to separate groups, thus eliminating the effect of individual differences. The second stage categorizes four emotions into two emotion pools in order to reduce recognition complexity. The third stage trains a classifier based on emotions in each emotion pool. During the testing process, a test case or test trial will be initially classified to a group followed by classification into an emotion pool in the second stage. An emotion will be assigned to the test trial in the final stage. In this paper we consider two different ways of allocating four emotions into two emotion pools. A comparative analysis is also carried out between the proposal and other methods.

**Results:**

An average recognition accuracy of 77.57% was achieved on the recognition of four emotions with the best accuracy of 86.67% to recognize the positive and excited emotion. Using differing ways of allocating four emotions into two emotion pools, we found there is a difference in the effectiveness of a classifier on learning each emotion. When compared to other methods, the proposed method demonstrates a significant improvement in recognizing four emotions in the multi-subject context.

**Conclusions:**

The proposed three-stage decision method solves a crucial issue which is ’individual differences’ in multi-subject emotion recognition and overcomes the suboptimal performance with respect to direct classification of multiple emotions. Our study supports the observation that the proposed method represents a promising methodology for recognizing multiple emotions in the multi-subject context.

## Background

Humans view the world through an individual perceptual filter and emotions (more accurately stated as *emotional response*) which are formed by a broad and diverse range of personality driven thoughts and behaviors. Emotive response is a psycho-physiological process triggered spontaneously by conscious and unconscious sensing of an object or context [1]. In human working and living environments, the expression and understanding of emotions helps achieve efficient intercommunication. With the exponential growth in human-computer interaction (HCI) applications, an accurate comprehension of emotion from a human perspective is required for a computer to achieve effective intercommunication with the triggering of proper feedback. In such HCI applications, human emotions can be delivered to computers in the form of either a subjective route by acquiring questionnaire responses of a subject or an objective route by measuring a subject’s emotional channels. The former approach is subjective and has some shortcomings which include selective reporting biases [2] and interference with real-time data collection [3]. Objective methods estimate emotions via human communication channels, such as speech, facial expression, gesture, pose and physiological responses [4–7]. However, speech, facial expression, gesture and pose are prone to deception, and may not be collected while subjects are in a natural and relaxed state. In contrast, various studies [7–10] show that physiological signals, such as electroencephalogram (EEG), electrocardiogram (ECG), electromyogram (EMG), galvanic skin response (GSR) and respiration (RSP), provide informative characteristics in response to emotions.

Haag et al. [11] employed a neural network classifier to classify positive *vs.* negative (two valence classes) and excited *vs.* clam (two arousal classes) respectively. The recognition accuracy reached 89.90% on valence dimension and 96.60% on arousal dimension. In their experiment, five physiological signals were collected when a single subject was exposed to visual stimuli. The five physiological signals include ECG, blood volume pulse (BVP), skin conductance (SC), EMG and RSP. Wagner et al. [7] achieved an accuracy of 92.05% for recognizing four emotions by combining four-channel bio-signals (EMG, ECG, SC and RSP) from a single subject. More recently, Huang et al. [12] extracted features from facial expression and EEG from 27 subjects. They fused these modalities and obtained the highest accuracy of 66.28% for valence and 63.22% for arousal by using decision-level fusion. Zhang et al. [13] collected RSP, EMG and SC from 20 subjects. They obtained the highest recognition rate of 80.00% for arousal and 76.67% for valence by combing these three modalities. When 4 classes were set by considering arousal and valence dimensions spontaneously, the highest accuracy of 50.00% was obtained by single modality RSP or EMG rather than modality fusion.

In well-documented works, ‘individual differences’ have been raised as a matter of widespread concern. Human beings may exhibit differing feelings upon a same emotion and have different physiological patterns when exposed to a same emotional stimulus. This issue was first proposed by Picard et al. [14]. The basic motivation for the use of physiological signals as opposed to subjective self-reports in emotion recognition was to discover the inner association of signal patterns with human emotional states and eventually to recognize emotions accurately. However, the classifier could not make an accurate judgment with respect to the context of multiple subjects when physiological patterns from different subjects exhibit large discrepancies upon a same emotion. Consequently, experimental subjects were limited to one person in numerous researches [7, 11, 14, 15]. However, other subjects cannot use this specific classification model that derived from one single subject.

Single-subject approaches have always been questioned as they fail in terms of universal applicability. Correspondingly, researches into emotion recognition [5, 7, 8, 15, 16] have kept a watchful eye on subject-independent way (which means a classification model built by physiological signals mixed across persons). It has been show that the subject-independent way usually behaves poorly as compared to the subject-dependent way (which means each classification model built by only a single person) owing to the effects of ‘individual differences’. Thus, the selection of either way may be viewed in terms of a tradeoff between universality and specificity. For the subject-independent case, research [8, 17, 18] has suggested that the recognition rate can be improved by transforming the subject-independent way to the subject-dependent way. Kim and André [8] merely offered this as a suggestion and they did not experimentally elaborate on this issue. Yuan et al. [17] and Gu et al. [18] used the physiological data of each subject to build separate classification models during the training process. During the testing phase, Yuan et al. [17] and Gu et al. [18] initially identified a classification model the test subject is assigned to and then performed emotion recognition by using the specific classification model. Their method approximates to a subject-dependent approach, but the feasibility is restricted to a limited number of subjects. For example, if there were one-thousand subjects, there would be one-thousand subject-specific models in their system resulting in significant computational overhead which may be unsuitable in real-world applications.

In this paper, we propose a novel three-stage decision method for multi-subject emotion recognition from physiological signals. The basic idea is to transform traditional subject-independent emotion recognition into group-dependent recognition. Specifically, in the initial stage of the training process, mixed training subjects are clustered into groups predicated on the characteristics of their physiological signals with the four emotions being allocated to two emotion pools in the second stage. Classifiers are trained in the third stage to classify two emotions in each emotion pools. During the testing phase, a test case/trial will be initially classified to a group in the first stage and classified to an emotion pool in the second stage. The exact emotion will be determined in the third stage. Details of the whole decision process will be elaborated in subsequent sections.

## Methods

### Dataset and feature extraction

#### Experimental data

The Database for Emotion Analysis using Physiological Signals (DEAP) [19] is being used in this study. It contains EEG, peripheral physiological signals and face videos from 32 subjects. The EEG signals were recorded from 32 active electrodes 32 channels according to international 10-20 system. Peripheral physiological signals (8 channels) include GSR, skin temperature (TMP), BVP, RSP, EMG collected from zygomaticus major and trapezius muscles, and EOG (horizontal and vertical). All of physiological signals were recorded while 40 (carefully selected) one-minute music clips were played in a random order to each subject, thus 40 trials per subject were generated. The DEAP dataset also contains self-reports of five dimensions of emotion (valence, arousal, dominance, liking and familiarity). The first four scales range from 1 to 9 and the fifth dimension ranges between 1 and 5. Among these dimensions, two dimensions represent various facets of emotion as follows:

*Valence*: ranging from negative (or unpleasant) to positive (or pleasant);

*Arousal*: ranging from calm (or bored) to active (or excited).

**Fig. 1:**
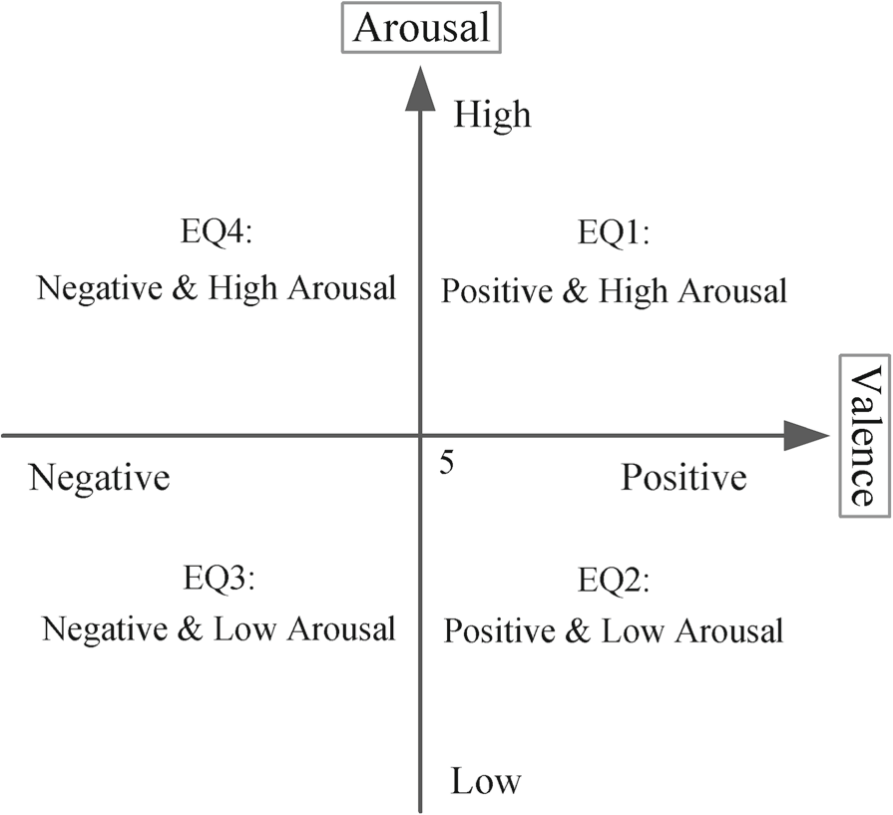
Valence-arousal space. EQ1: valence rating > 5 and arousal rating > 5, EQ2: valence rating > 5 and arousal rating ≤ 5, EQ3: valence rating ≤ 5 and arousal rating ≤ 5, EQ4: valence rating ≤ 5 and arousal rating > 5

Each discrete emotional state (for example, joy, sadness and anger, etc.) can be placed to the two-dimensional valence-arousal space [20, 21]. The two-dimensional valence-arousal space is shown in Fig. 1. A full description of the database is shown in Table 1.

**Table 1:** Database content summary

Online subjective annotation	
Number of videos	120
Video duration	1 min affective highlight
Selection method	60 via last.fm affective tags, 60 manually selected
Number of ratings per video	14–16
Rating scales	Arousal, Valence and Dominance
Rating values	1–9
Physiological experiment	
Number of subjects	32
Number of videos	40
Selection method	Subset of online annotated videos with clearest responses
Rating scales	Arousal, Valence, Dominance, Liking and Familiarity
Rating values	Familiarity: discrete scale of 1-5, others: continuous scale of 1-9
Recorded signals	32-channel 512Hz EEG, peripheral physiological signals, face video from 22 subjects

In this study, we use two basic emotion dimensions: valence and arousal. In 40 stimuli/trials, 17 stimuli are labeled with discrete emotional states while others have no emotional labels. Therefore, 544 trials labeled with discrete emotional states and collected from 32 subjects have been used in this study. The subjects’ arousal and valence self-ratings are used as the ground truth.

Our aim is the classification of four emotions (EQ1, EQ2, EQ3 and EQ4) corresponding to four quadrants shown in Fig. 1 where the discrete emotional states of the 544 trials are mapped.

#### Feature extraction

Table 2 provides a complete overview of extracted features. The total number of extracted features from all physiological channels is 742 and the power spectral features are calculated by fast Fourier transform (FFT).

**Table 2:** Features extracted from physiological signals

Feature index	Notation of the extracted features
No. 1-448 EEG time and frequency-domain features (14 feature types × 32 channels)	Mean, Var, peak-to-peak amplitude, Skewness, Kurtosis
	Average PSD in theta (4-7 Hz), alpha (8-15 Hz),beta (16-31 Hz), gamma (32-45 Hz), beta/theta, beta/alpha
	Three Hjorth parameters: mobility, activity and complexity
No. 449-504 EEG hemispheric asymmetry (4 feature types × 14 channel pairs)	Difference of average PSD in theta, alpha, beta and gamma bands for 14 channel pairs between right and left scalp
No. 505-600 EEG nonlinear features (3 feature types × 32 channels)	Spectral Entropy, Shannon Entropy and C0 complexity
No. 601-608 EOG features (4 feature types × 2 channels)	Mean, Var, peak-to-peak amplitude, Energy
No. 609-642 EMG features (17 feature types × 2 channels)	Mean, Var, Total spectral power
	1Diff-Mean, 1Diff-Median, 1Diff-Min, 1Diff-Var, 1Diff-Max, 1Diff-MinRatio, 1Diff-MaxRatio
	2Diff-Mean, 2Diff-Median, 2Diff-Min, 2Diff-Var, 2Diff-Max, 2Diff-MinRatio, 2Diff-MaxRatio
No. 643-646 TMP features (4 feature types × 1 channel)	Mean, 1Diff-Mean, Spectral power in the bands (0-0.1 Hz) and (0.1-0.2 Hz)
No. 647-666 BVP features (20 feature types × 1 channel)	Hr-Mean, Hr-Var, Hr-Range
	Hrv-Mean, Hrv-Var, Hrv-Min, Hrv-Max, Hrv-Range, Hrv-pNN50
	HrvDistr-Mean, HrvDistr-Median, HrvDistr-Var, HrvDistr-Min, HrvDistr-Max, HrvDistr-Range, HrvDistr-Triind
	PSD in bands (0-0.2 Hz), (0.2-0.4 Hz), (0.4-0.6 Hz), and (0.6-0.8 Hz) of Hrv
No. 667-721 RSP features (55 feature types × 1 channel)	Mean, Var, Range, MaxRatio
	1Diff-Mean, 1Diff-Median, 1Diff-Var, 1Diff-Range, 1Diff-MaxRatio
	2Diff-Mean, 2Diff-Median, 2Diff-Var, 2Diff-Range, 2Diff-MaxRatio
	RSPPulse-Mean, RSPPulse-Var, RSPPulse-Range, RSPPulse-MaxRatio
	RSPPulse-1Diff-Mean, RSPPulse-1Diff-Median, RSPPulse-1Diff-Var, RSPPulse-1Diff-Min, RSPPulse-1Diff-Max, RSPPulse-1Diff-Range, RSPPulse-1Diff-MaxRatio
	RSPPulse-2Diff-Mean, RSPPulse-2Diff-Median, RSPPulse-2Diff-Var, RSPPulse-2Diff-Min, RSPPulse-2Diff-Max, RSPPulse-2Diff-Range, RSPPulse-2Diff-MaxRatio
	RSPAmpl-Mean, RSPAmpl-Var, RSPAmpl-Range, RSPAmpl-MaxRatio
	RSPAmpl-1Diff-Mean, RSPAmpl-1Diff-Median, RSPAmpl-1Diff-Var, RSPAmpl-1Diff-Min, RSPAmpl-1Diff-Max, RSPAmpl-1Diff-Range, RSPAmpl-1Diff-MaxRatio
	RSPAmpl-2Diff-Mean, RSPAmpl-2Diff-Median, RSPAmpl-2Diff-Var, RSPAmpl-2Diff-Min, RSPAmpl-2Diff-Max, RSPAmpl-2Diff-Range, RSPAmpl-2Diff-MaxRatio
	PSD in the bands (0-0.1 Hz), (0.1-0.2 Hz), (0.2-0.3 Hz), and (0.3-0.4 Hz), Ratio of PSD in the band (0-0.25 Hz) to PSD in the band (0.25-0.45 Hz)
No. 722-742 GSR features (21 feature types × 1 channel)	Rising time, Decay time
	Sc-Mean, Sc-Median, Sc-Var, Sc-MinRatio, Sc-MaxRatio
	Sc-1Diff-Mean, Sc-1Diff-Median, Sc-1Diff-Var, Sc-1Diff-Min, Sc-1Diff-Max, Sc-1Diff-MinRatio, Sc-1Diff-MaxRatio
	Sc-2Diff-Mean, Sc-2Diff-Median, Sc-2Diff-Var, Sc-2Diff-Min, Sc-2Diff-Max, Sc-2Diff-MinRatio, Sc-2Diff-MaxRatio

MaxRatio: number of maxima divided by the total number of signal values, MinRatio: number of minima divided by the total number of signal values, 1Diff: approximation of first derivation, 2Diff: approximation of second derivation, Range: maximum-minimum, RSPPulse: pulse signal of RSP, RSPAmpl: amplitude signal of RSP, Sc: skin conductance, Hr: heart rate, Hrv: heart rate variability, pNN50: number of pairs of adjacent NN intervals differing by more than 50ms in the entire recording divided by the total number of NN intervals, HrvDistr: distribution of NN intervals, HrvDistr-Triind: total number of all NN intervals divided by the height of the histogram of all NN intervals, Var: variance, PSD: power spectral density

Each trial lasts one minute, but a subject’s physiological patterns might not be consistent with the emotional content of a stimulus for one entire minute. Therefore, features are extracted with 4-second sliding and 2-second overlapped time windows. This is in contrast to traditional extraction way in which one sample is extracted from a trial. In our research, each trail is represented by 29 samples, thus we obtain a feature matrix with the size of 15576×742 (samples ×features), where 15576 samples are equal to 32 subjects × 17 trials/subject × 29 samples/trial.

Each column of the feature matrix is normalized to [0,1]. These normalized features are the input to the whole decision method. In each stage of the proposed method, Fisher Criterion Score (FCS) [22] is performed on the feature matrix to rank features. FCS attempts to find a feature subset where samples from the same class are assembled, whereas samples from different classes are separated to the maximum level:

$$F_{l}=\frac{(m_{1,l} - m_{2,l})^{2}}{\sigma^{2}_{1,l} + \sigma^{2}_{2,l}}. $$

where the mean and standard deviation of samples belonging to two classes *C* *l* *a* *s* *s*_1_ and *C* *l* *a* *s* *s*_2_ are denoted by *m*_1,*l*_, *m*_2,*l*_, *σ*_1,*l*_ and *σ*_2,*l*_ respectively for the *l*-th feature (*l*=1,2,…,742). *F*_*l*_ denotes the capability of the *l*-th feature to separate two classes. Thus, a feature list sorted by all *F*_*l*_ values in descending order is obtained. The first feature is the most relevant, while the last one is the least important.

### The three-stage decision method

**Fig. 2:**
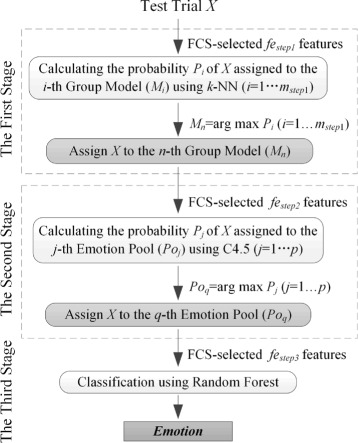
Diagram of the three-stage decision method for multi-subject emotion recognition

The basic idea of the proposed method is the transformation of a general subject-independent emotion recognition into a group-dependent recognition by classifying a test trial into a group model prior to the emotion recognition procedure. The steps described in the following sections create our decision method (shown in Fig. 2) for multi-subject emotion recognition. In the first stage, group models are built by categorizing training subjects during a training process. In a testing phase, a test trial will be classified to a group model. In the second stage, emotion pools are built for each group model during the training process, an emotion pool being made up of two emotions. During the testing phase, the test trial will be assigned to a particular emotion pool. In the third stage, an explicit emotion will be assigned to the test trial during the testing process. Based on the foregoing process, the first stage can be regarded as subject partitioning, the second stage as the classification of emotion pools, and the third stage as two-emotion classification.

A test trial is classified to a group model *M*_*n*_ or an emotion pool *P* *o*_*q*_, the process is reliant on the maximum probability that the test is classified to the model *M*_*n*_ or the pool *P* *o*_*q*_. The decision process then proceeds to the third stage which performs two-emotion classification.

All of three components are described in the following sections.

#### The first stage

During the training phase, the objective of the first stage is to build group models *M*_1_,*M*_2_,…,*M*_*m*_ from the training subjects *S*_1_,*S*_2_,…,*S*_*c*_ (*m*≪*c*, *c*=31). Each group model is built by a feature set of a subject group that is disjoint from other groups.

Given that 742 features may not be all relevant to the subject partitioning, *f* *e*_*step*1_ features are selected to cluster *c* subjects to *m* groups. As this stage aims to categorize subjects with the next two stages classifying emotions, features used in this stage should be selected in the context of both the subject partitioning and the emotion classification. We use the following steps to obtain *f* *e*_*step*1_ features:

1. We assemble *c* subjects’ feature matrices which are labeled with an emotion *s* (*s*=EQ1, EQ2, EQ3 or EQ4) and take subject IDs as classification labels. Figure 3 presents graphically an illustration of combining together subjects’ feature matrices labeled with EQ1. We then label the generated feature matrix (shown in Fig. 3(b)) with subject IDs (e.g., ’S1’, ’S2’,…, ’S31’). Finally, a ranked feature list is generated from the generated feature matrix with emotion EQ1 by using FCS ranking approach. The first feature in the list is viewed as the most significant and the last feature as the most irrelevant feature in subject partitioning.

**Fig. 3:**
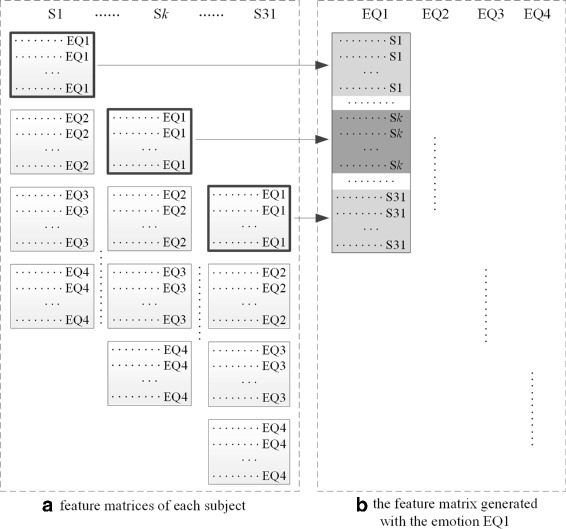
The process of feature selection in stage one. ’ *S*_*k*_’ (*k*=1,2, …,31) is subject IDs
2. Four ranked feature lists are obtained respectively for the four emotions.
3. We take out first *f* *e*_1_ features from each ranked feature list, where *f* *e*_1_ is a variable ranging from 1 to 742 with a step of 5. When a value is tested, the intersection of the four *f* *e*_1_-sized feature subsets is denoted as a feature set *F* *S*_1_. The feature size in *F* *S*_1_ varies with the change of *f* *e*_1_.
4. Thirty-one subjects are classified by using each feature set *F* *S*_1_. Classification performance of using different *F* *S*_1_s is compared and when a classification performance peaks, the corresponding feature set will be the optimal one.
5. When this optimal feature set is identified, we use *k*-means clustering algorithm to cluster *c* subjects to *m* groups, where *m* is a variable ranging from 2 to 5. As the physiological data of one subject (29 samples/trial ×17 trials = 493 samples) may not be mapped to one cluster completely, the subject will be assigned to the group to which most of the subject’s data are clustered. We label 15283 samples (=31 subjects × 17 trials/subject × 29 samples/trial) with *m* group models according to the clustering result and the data distribution among group models is observed. For example, when we test *m*=4, if samples are distributed extremely unevenly among group models, we will not consider *m*=4 any more and the next *m* value (*m*=5) will be tested. Otherwise, if the data are distributed relatively evenly among group models, *m* (=4) classification models will be built and we classify a validation set to these 4 classification models. Finally, the classification performance of using different *m* values is compared. When the classification performance of using a particular value peaks, the optimal *m* value is identified, which is denoted as *m*_*step*1_.
6. While the optimal feature set generated in step 4 performs well in subject classification, the optimal feature set for clustering subjects into groups should be further identified. We use FCS approach to rank 742 features and take out the first *f* *e*_*s*1_ features to classify *m*_*step*1_ group models, where *f* *e*_*s*1_ is a variable ranging from 1 to 742. When a classification performance peaks, the optimal *f* *e*_*s*1_ value will be identified as *f* *e*_*step*1_. Based to the foregoing process, the optimal feature set *f* *e*_*step*1_ and the number of group models *m*_*step*1_ are identified.

During the testing phase, a test trial *X* (a feature matrix with size of 29 samples × 742 features) will be classified to a group model. The decision process is described as below:

1. Calculating the probability *P*_*i*_ that *X* is assigned to the *i*-th group model *M*_*i*_ (*i*=1…*m*_*step*1_) using *k*-Nearest-Neighbor (*k*-NN) algorithm;
2. Identifying the *n*-th group model *M*_*n*_ that gives the maximum probability for *X*;
3. Assigning *X* to the corresponding group model *M*_*n*_.

Given that the sum of probabilities that one sample is assigned to every group model is equal to one, the sum of probabilities of the test trial *X* assigned to every group model is equal to 29. Thus, the sum of probabilities of a test trial assigned to a group model ranges in [0,29].

In this first stage, *k*-NN algorithm is used for assigning the test trial *X* to a particular group model *M*_*i*_. According to *k*-NN algorithm, if there are more samples of the group model *M*_*i*_ among *k* nearest neighbors of a test sample, *k*-NN will assign the group model *M*_*i*_ to the test sample. A more detailed description of the algorithm can be found in [23]. The probability of a sample assigned to a group model *M*_*i*_ is calculated by the number of samples of the model *M*_*i*_ in *k* nearest neighbors divided by *k* neighbors. Accordingly, we can obtain the sum of probabilities of the test trial *X* assigned to a particular group model.

#### The second stage

In this stage, the aim is to transform the recognition of multiple emotions to the recognition of emotion pools. Emotion pools are built for each group model. Given a group model, emotions in an emotion pool are disjoint from those in other emotion pools. In this study, we test 2 combination ways: (a) putting EQ1 and EQ4 in a pool, EQ2 and EQ3 in another pool, and (b) mixing EQ1 with EQ2 in a pool, EQ3 and EQ4 in another pool. In this section, we detail the first combination way and the other way has the same process. As shown in Fig. 1, two emotion pools in the first combination way actually correspond to high arousal (HA) and low arousal (LA), while emotion pools in the second combination way correspond to high valence (HV) and low valence (LV).

During the training phase, the physiological data of both EQ1 and EQ4 are used to train the first emotion pool (*P* *o*_1_) and the data from both EQ2 and EQ3 used to train the second emotion pool (*P* *o*_2_). Initially, FCS feature ranking approach is used in each group model where the target variables are two emotion pools. For each group model, we take out the first *f* *e*_2_ features from its ranked feature list where *f* *e*_2_ is a variable ranging from 1 to 742. We test each *f* *e*_2_-sized feature set on a validation set. When a classification performance peaks, the optimal *f* *e*_2_ value is denoted as *f* *e*_*step*2_. It is noted that each group model generates an optimal feature set.

During the testing phase, following assignment of the test trial *X* to the *n*-th group model *M*_*n*_ in the first stage, the original subject-independent emotion recognition is transformed to the group-dependent recognition. In the second stage, we assign the test trial *X* to an emotion pool and in the third stage an explicit emotional state will be identified for *X*. The test trial *X* is assigned to an emotion pool *P* *o*_*q*_ by the following steps:

1. Calculating the probability *P*_*j*_ that *X* is assigned to the *j*-th emotion pool *P* *o*_*j*_ (*j*=1,2) using C4.5 decision tree algorithm;
2. Identifying the *q*-th emotion pool *P* *o*_*q*_ that gives the maximum probability for *X*;
3. Assigning *X* to the corresponding emotion pool *P* *o*_*q*_.

In the stage two, C4.5 decision tree is used to assign the test trial *X* to an emotion pool. C4.5 algorithm applies information gain ratio to generate a decision tree whose leaf nodes represent two emotion pools and inner nodes represent features. In each split of the tree, C4.5 algorithm calculates information gain ratio of each feature and applies the most robust features to construct the tree. FCS approach used in the second stage is merely to decrease the number of features which are redundant but would have been calculated in the construction of a tree. A detailed exposition on C4.5 algorithm is provided by Quinlan [24]. The probability of a test sample assigned to a specific emotion pool *P* *o*_*q*_ is calculated as follows: (a) starting from the root node, the probability distribution of classes in each node that the sample goes through are calculated; (b) all probabilities calculated for the class *P* *o*_1_ are added up and all probabilities calculated for the class *P* *o*_2_ are added up; (c) two probability values are normalized; and (d) the test sample is assigned to the emotion pool *P* *o*_*q*_ with the higher probability. Accordingly, we can obtain the sum of probabilities that the test trial *X* is assigned to a particular emotion pool.

#### The third stage

In this stage, a particular emotion is finally identified for the test trial *X* after the trial assigned to the group model *M*_*n*_ and the emotion pool *P* *o*_*q*_.

During the training phase, we initially rank the features using FCS feature ranking approach. We then take out the first *f* *e*_3_ features, where *f* *e*_3_ is a variable ranging from 1 to 742. Given a candidate value, we obtain a *f* *e*_3_-sized feature set. Using this feature set, we classify a validation set to two emotions by using Random Forest (RF) algorithm. When the classification performance of using a particular *f* *e*_3_ value peaks, the optimal *f* *e*_3_ value is identified, which is denoted as *f* *e*_*step*3_.

During the testing process, after the test trial is classified to the emotion pool *P* *o*_*q*_ in the second stage, the classification in the third stage is performed as follows:

1. Calculating the sample number in the test trial *X* that are classified into each of the two emotions;
2. Assigning *X* to the emotion to which the majority of the samples in *X* are classified.

Random Forest is applied in this stage. It is recognized as one of the most prominent techniques of emotion classification. Random Forest is similar to C4.5 with respect to the composition aspect. A random forest is made up of many random trees. When a random forest is applied to a test sample, a decision is obtained by the majority voting received from all random trees. We can obtain the decision for a sample, thus the decision result for the test trial *X* can be also achieved. The detailed description of the Random Forest can be found in [25].

## Results

### Data partitioning during the training process

We have used a leave-one-subject-out cross-validation method, where a single subject taken from the whole dataset is used as the test subject while the remaining dataset is used in the training process. This cross-validation process is iteratively repeated until each subject is used as the test subject once. Therefore, there are 31 subjects in the training process and one subject in the testing process in each loop of the cross-validation process. In order to identify the optimal feature set and classifier parameters during the training process, we further separated the data of 31 subjects into a training set and a validation set. In each loop of the cross-validation process, we built classification models using a training set with different classifier parameters and tested these classification models on a validation set. When a classification performance peaks, the corresponding parameters and the feature set are the optimal ones.

In the first stage, both subject partitioning and four emotions are taken into account. Since the optimal parameters are identified based on the classification result of the validation set, we included 31 trials in the validation set, each trial from one subject. These 31 trials also include four emotions. If one subject has only one trial with a particular emotion, this trial will be allocated to the training set for classifiers to learn. In this stage, the number of group models (*m*) was also identified. When the number of group models was set to 2, 31 subjects were allocated relatively evenly to two groups. When the number of group models was set to 3 to 5 in each loop of the cross-validation process, most subjects were assigned to the first or the second group while only 1 or 2 subjects were assigned to the other groups. If there was only one or two subjects in a group, there would be limited information for a classifier to learn. Therefore, the optimal *m* (*m*_*step*1_) was set to 2 in each loop of the cross-validation.

In the second stage, we created a training set and a validation set for each group model. In each group model, if one subject has more than 5 trials of a particular emotion, we randomly chose one trial into a validation set. It is impossible for a subject to have every emotion with more than 5 trials; therefore, the validation set would not completely come from one subject. In other words, the validation set includes 4 emotions and all of trials in the validation set will not come from a single subject.

In the third stage, we also obtained a training set and a validation set from each emotion pool. There are two emotions in an emotion pool. To evaluate the performance in classifying each emotion, a validation set should include these two emotions. If one subject has more than 4 trials of a particular emotion (according to his self-reports), we randomly chose one trial into a validation set. If no subject has a particular emotion in more than 4 trials, then we will search for subjects who have that emotion in more than 3 trials.

### Optimal features and classifier parameters identified during the training process

In each stage of the proposed method, we tested four classifiers: *k*-NN, C4.5, RF and SVM. The classifier parameters, such as *k* in *k*-NN, SVM kernels including linear, polynomial, sigmoid and radial basis function (RBF), have also been investigated.

**Fig. 4:**
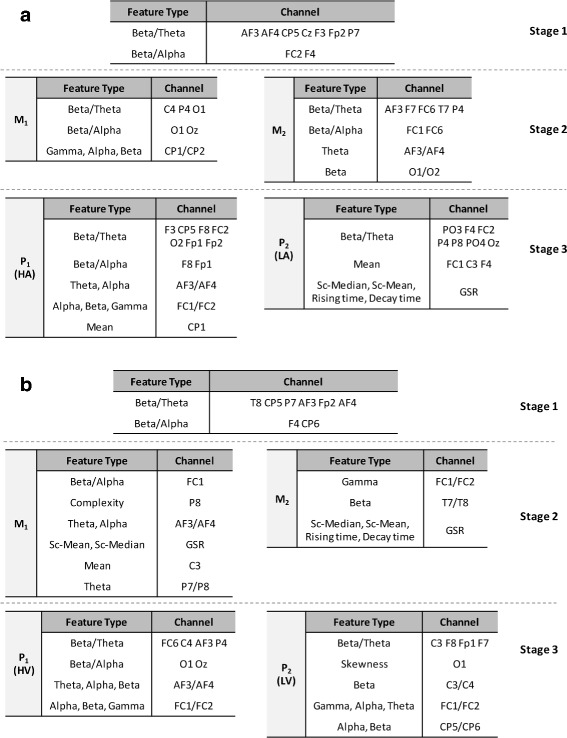
Most selected features in each stage. **a** Most selected features in each stage when two emotion pools corresponding to HA and LA. **b** Most selected features in each stage when two emotion pools corresponding to HV and LV

In each loop of the leave-one-subject-out cross-validation, four classifiers achieved the same recognition capability in the first stage. All of these classifiers achieved the recognition accuracy of 100% when tested on validation sets. The *k*-NN proved to be faster than other classifiers, for this reason we chose it in the first stage. As we used the training data and the validation data differently in each loop, the optimal features selected in each loop are not the same. The most frequently selected features are shown in Fig. 4. Following the first stage, there are two groups *M*_1_ and *M*_2_. Most training subjects are allocated to the first group *M*_1_, while the 7th and 23rd subjects (, sometimes grouped with few other subjects during some loops) are allocated to the second group *M*_2_. If the 7th or 23rd subject is the test subject, it will not appear in the second group during the training process.

In the second stage, C4.5 outperformed other classifiers in classifying two emotion pools in each group model, but the classifier parameters were set differently in each loop of the cross-validation. For each group model, an optimal feature set was generated to classify two emotion pools. The most selected features are listed in Fig. 4. Figure 4a shows the most selected features in three stages when constructing two emotion pools as HA and LA, while Fig. 4b shows features when constructing two emotion pools as HV and LV.

In the third stage, RF performed better than other classifiers on recognizing two emotions in each emotion pool. Since the emotions in two pools are different, the optimal feature sets are also different between two pools. The most selected features for two emotion pools are listed separately in Fig. 4.

From Fig. 4, it can be seen that EEG, specifically, the features of EEG hemispheric asymmetry and power ratio, shows its importance in emotion recognition as well as subject partitioning. In both Fig. 4a and b, GSR also shows its superiority to emotion classification.

### Decision results in each stage

Table 3 summarizes the classification results with respect to the stage-divided strategy used in our decision method. Creating two emotion pools as HA and LA, the results show that the emotion EQ1 is well learnt with a classification accuracy of 86.67%, whereas there is evidence of misclassification

**Table 3:** Recognition performance of allocating four emotions to two emotion pools differently

Strategy	Recognition accuracy				
	EQ1	EQ2	EQ3	EQ4	Average
Two pools: HA and LA	86.67%	80.00%	30.56%	58.33%	77.57%
Two pools: HV and LV	33.33%	83.33%	50.55%	50.00%	43.57%

for EQ3 as demonstrated by a classification accuracy of 30.56%. When constructing two emotion pools as HV and LV, the classification of EQ2 is better than for the other emotions. The best classification rate reaches 83.33%, but the classification performance does not exceed 55.00% for the three other emotions. Comparing two ways of allocating four emotions to two pools, we found that different allocations lead to different ability of a classifier learning each emotion. The average recognition rate of the whole dataset is 77.57% when forming emotion pools as HA and LA. The average recognition rate is 43.57% when forming emotion pools as HV and LV. We also calculated the subject agreement on four emotions based on their self-reports, we found the mean agreement of 75.00% across 32 subjects. It can be seen that subjects frequently disagreed on the affective content of videos. The subjects found it hard to reach an agreement on the affective content, thus it may be harder for machines to recognize human emotions 100%.

In this study, a MATLAB toolkit, integrating diverse machine learning algorithms, known as Waikato Environment for Knowledge Analysis (Weka), is used to perform classification in each stage. We also use Augsburg Biosignal Toolbox (AuBT) to extract features from physiological signals except EEG. The decision methodologies, their modifications and the whole analysis code developed for the decision method were implemented in MATLAB R2016b.

## Discussion

### A comparison with other multiclass classification methods

In this study, we attempt to recognize four emotions from multi-subject physiological signals. The encouraging recognition results are obtained using the three-stage decision method with multiple samples representing a trial.

We also tested a number of methods widely used in the recognition of multiple emotions:

1. *k*-NN and SVM, two popular classifiers, capable of directly performing multiclass classification. We used one sample extracted from one trial as the classifier input, thus we obtained a feature set with 544 samples (=32 subjects × 17 trials).
2. One-against-Rest scheme. In this method, one emotion is regarded as one single class and the rest three emotions are regarded as one mixed class. A classifier is applied in each binary classification. Thus, we trained $C_{4}^{1}=4$ classifiers in total.
3. One-against-One scheme. In this method, a classifier is applied in a binary classification task in which two of the four emotions are taken out as two classes. Thus, $C_{4}^{2}=6$ classifiers were trained.

**Fig. 5:**
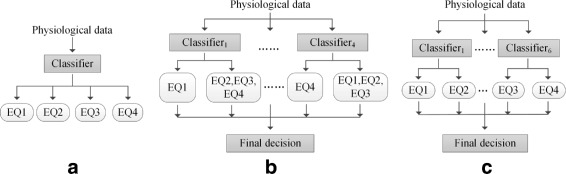
Three typical multiclass classification ways. **a** Multiclass classifiers. **b** One-against-Rest scheme. **c** One-against-One scheme

A simple illustration of the above comparative methods can be seen in Fig. 5. A comparison of these methods with our proposed method is shown in Table 4 with a detailed description. The leave-one-subject-out cross-validation was exploited in all the above methods. For One-against-Rest and One-against-One schemes respectively, a final decision was made based on outputs of every classifier. Two typical decision fusion approaches, majority voting and sum rule [1, 26], were used separately to derive final decisions. Majority voting approach sums up classifiers’ decisions for each class. Sum rule approach sums up the support values generated by classifiers for each class. Finally, the class obtaining more decisions or a higher support value is the final decision.

**Table 4:** Parameter setting and recognition performance of comparative methods

	Method	Parameters	Description	Accuracy
1	*k*-NN	*k*=1		44.30%
	SVM	Linear kernel, cost C=200, tolerance E=0.001, epsilon for the loss function P=1.0E-12	One sample from one trial	51.10%
	C4.5	-		47.24%
	Random forest	-		46.69%
2	One-against-Rest	*k*-NN, majority voting	29 samples from one trial	51.13%
3	One-against-One	*k*-NN, majority voting		52.16%
	Three-stage decision	Two pools: HA and LA		77.57%

It can be seen from the results shown in Table 4 that when one sample calculated from one trial was used, SVM outperforms *k*-NN in recognizing four emotions with the recognition accuracies of 51.10% and 44.30% respectively. The classifier C4.5 obtained the recognition accuracy of 47.24% which is slightly higher than RF with the accuracy of 46.69%. However, the classifier *k*-NN outperforms other classifiers when used in both One-against-Rest and One-against-One schemes, and the results of using *k*-NN are listed in the table. The best accuracies achieved by both schemes are 51.13% and 52.16% respectively with the decision fusion approach of majority voting used. For One-against-Rest scheme, each mixed class involves three emotions plus individual differences, meaning that it contains wide range of physiological data which may cover partial data of the single class. This may be a reason for the low recognition rate of 51.13%. For One-against-One scheme, for example, a classifier *C* is trained by a training set labeled with both EQ2 and EQ3 emotions and a test trial is labeled as EQ1. When the classifier *C* is applied to classify the test trial, the decision will be completely wrong irrespective of the class to which the trial is classified. The rationale for this observation is that because the real emotion (EQ1) of the test trial is totally different from the emotions (EQ2 and EQ3) of the training set and the decision reached by the classifier must be either EQ2 or EQ3 rather than EQ1. This tupe of incorrect decisions may lead to the low recognition rate of 52.16% for One-against-One scheme.

The proposed method incorporates the capability to deal with the problems inherent in the above methods. Specifically, we exploited multiple samples calculated from a trial rather than the traditional manner of one sample calculated from a trial. One sample might be deficient in describing the emotional information contained in a video stimulus. This can be also explained by the phenomenon in which human physiological response to a stimulus is transient and may be rarely consistent with an emotion task for one entire minute. Furthermore, our approach eliminates the effect of individual differences and reduces substantial incorrect decisions arising in One-against-Rest and One-against-One schemes.

### Limitations and future work

While our research has addressed a number of existed problems, there are perceived limitations in our method. Firstly, we employed a traditional feature fusion approach which is the direct concatenation of normalized channel features. Combining multiple modalities by equally weighting them does not always guarantee satisfactory accuracy. Recent studies [1, 26–28] have explored multimodal information fusion techniques to enable emotion recognition. Two categories of multimodal information fusion are: feature fusion and decision fusion. In this paper, two approaches of decision fusion have been investigated (see “Discussion” section), but the results obtained by using them in One-against-Rest and One-against-One schemes are not promising. In considering feature fusion, a crucial issue is how to combine supplementary and complementary data from different modalities. It may help improve the recognition performance when advanced multimodal fusion techniques are exploited in our method.

Secondly, we adopted subjects’ self-ratings to label emotion classes. This may lead to a negative impact on recognition results, since subjects held different feelings of the affective content in videos. We can see that an agreement of 75.00% was given by 32 subjects. Therefore, a more appropriate labeling strategy is needed for our future study to guarantee the validity of the recognition results and to improve recognition rate.

Thirdly, we found different channels and feature types used in each stage, thus a great number of features should be filtered three times. In addition, the recognition results of the three stages synthesized the final result. The misclassification in first two stages may influence the classification in the third stage. Therefore, we should reference additional studies, such as identity recognition techniques, to find a robust but limited number of features to eliminate the decision errors in the first stage.

Finally, it is worthy of note that in real-world applications, physiological responses to stimuli may differ from responses collected in a well-controlled laboratory environment. In laboratory experiments, subjects were given a set of instructions and encouraged to give strongest feelings about the video stimuli. However, in real-world applications it is unlikely that any instructions or prompts would be provided. Given these observations, the presented results may overestimate the real recognition rates to some extent.

## Conclusions

In this paper, we proposed a three-stage decision method for subject-independent emotion recognition from physiological signals. To eliminate the poor recognition outcomes caused by ’individual differences’, we initially classify a test trial to a particular group model and then perform emotion classification in a group-dependent way. The best accuracy of 77.57% was achieved to recognize four emotions with accuracies of 86.67% on EQ1, 80.00% on EQ2, 30.56% on EQ3, and 58.33% on EQ4 respectively. The best classification result was derived by combining EQ1 with EQ4 to an emotion pool and mixing EQ2 and EQ3 into the other emotion pool. We also tested an alternative allocation way and compared our method with other multiclass classification methods. The improved results presented in this paper demonstrate the effectiveness of the three-stage decision method in emotion recognition.

In this study, a wide range of signal features calculated from various analysis domains, including time-frequency domain, entropy and complexity, were exploited to explore significant features in each stage. The most selected features were described in detail. We found that EEG modality is generally dominant for emotion differentiation since EEG features were employed in each of the three stages.

Considering the complex processing procedure involved in subject-independent emotion recognition, we could conclude that research on subject-independent emotion recognition using physiological signals represents a potentially fruitful and profitable direction for future research.

## Abbreviations

BVP: Blood volume pulse
DEAP: Database for emotion analysis using physiological signals
ECG: Electrocardiogram
EEG: Electroencephalogram
EMG: Electromyogram
EOG: Electrooculogram
FCS: Fisher Criterion Score
FFT: Fast Fourier transform
GSR: Galvanic skin response
HA: High arousal
HCI: Human-computer interaction
HR: Heart rate
HV: High valence
*k*-NN: *k*-Nearest-Neighbor
LA: Low arousal
LV: Low valence
RBF: Radial basis function
RF: Random forest
RSP: Respiration
SC: Skin conductance
SVM: Support vector machine
TMP: Skin temperature
Weka: Waikato environment for knowledge analysis
